# The Central Role of IFI204 in IFN-β Release and Autophagy Activation during *Mycobacterium bovis* Infection

**DOI:** 10.3389/fcimb.2017.00169

**Published:** 2017-05-05

**Authors:** Liu Chunfa, Sun Xin, Li Qiang, Srinand Sreevatsan, Lifeng Yang, Deming Zhao, Xiangmei Zhou

**Affiliations:** ^1^State Key Laboratories for Agrobiotechnology, Key Laboratory of Animal Epidemiology and Zoonosis, Ministry of Agriculture, National Animal Transmissible Spongiform Encephalopathy Laboratory, College of Veterinary Medicine, China Agricultural UniversityBeijing, China; ^2^Department of Veterinary Medicine, College of Agriculture, Ningxia UniversityYinchuan, China; ^3^Department of Veterinary Population Medicine, College of Veterinary Medicine, University of MinnesotaSaint Paul, MN, USA

**Keywords:** IFI204, IFN-β, *M. bovis*, autophagy, DNA sensor

## Abstract

*Mycobacterium bovis* (*M. bovis*) is the pathogen of animals and humans that can replicate in the phagosomes of myeloid cells. Cytosolic detection of bacterial products plays a crucial role in initiating the innate immune response, including autophagy activation and interferon-β (IFN-β) release. Although IFN-β release and autophagy activation have been reported during mycobacterium infection, the mechanisms underlying remains poorly defined. Here, we demonstrated that IFN-β release increases in macrophages exposed to *M. bovis* and this requires the activation of the DNA sensor of interferon-γ inducible protein 204 (IFI204). Knockdown of the IFI204 in immortalized and primary murine macrophages blocked IFN-β production and autophagy marker LC3 expression. Thus, our results indicate that the IFI204 is an important sensor for innate immune responses of *M. bovis* infection.

## Introduction

Tuberculosis (TB) is a major burden on global health, owing to its high morbidity and mortality worldwide. In 2010, 9 million new cases were reported that was associated with mortality of 1.4 million humans (O'Garra et al., 2013). The disease is caused by *Mycobacterium tuberculosis* (*Mtb*) and/or *M. bovis*. A recent review on the epidemiology of human TB caused by *M. bovis* in the United States reported that the annual percentages of TB cases attributable to *M. bovis* ranged from 1.3 to 1.6% during the period 2006–2013 (Scott et al., 2016). One constraint of the study was that a fraction (23.6%) of TB patients were not cultured and a proportion (15.5%) of the cultured isolates were not typed using molecular markers. Since *M. bovis* is pyruvate dependent and conventional culturing media does not contain it, this could have underestimated the burden of *M. bovis* (Scott et al., 2016).

Foreign DNA is detected by the innate immune system and triggers early protective responses. When lysed mycobacteria are released from phagosome into the cytoplasm, naked mycobacterial genomic DNA induces cytokine release and activate autophagy (Manzanillo et al., 2012; Watson et al., 2012). The proposed DNA sensors such as, absent in melanoma 2 (AIM2) (Saiga et al., 2012; Yang et al., 2013), cyclic GMP-AMP (cGAMP) synthetase (cGAS) (Collins et al., 2015; Wassermann et al., 2015; Watson et al., 2015) and IFI204 (Unterholzner et al., 2010; Manzanillo et al., 2012) participate in the recognition of bacterial DNA. Upon sensing cytosolic DNA, AIM2 protein recruits ASC (the adaptor molecule apoptosis speck like protein containing a CARD domain) and caspase-1 to up-regulate the secretion of IL-1β, while the cGAS protein produces a cellular secondary messenger-cGAMP which directly binds to the STING (adaptor the protein stimulator of IFN genes) leading to co-localization of LC3 with *Mtb* and phosphorylation of transcription factor IFN regulator factor (IRF3) that translocates into the nucleus to stimulate the type-I IFN expression. Although, IFI204 has been shown to contribute to the IFIT1 and IFN-β induction during *Mtb* infection (Manzanillo et al., 2012), the precise mechanism is not fully understood to date.

IFI204 (the murine ortholog of human IFI16) is a member of the interferon-inducible p200 family proteins (also known as PYHIN or HIN-200 proteins) that contains a PYHIN domain and two DNA binding HIN domains. During Kaposi sarcoma-associated herpesvirus (KSHV) infection of endothelial cells, IFI16 interacts with ASC and procapase-1 to form an inflammasome in IL-1β release (Kerur et al., 2011). The translocation of IFI16 from nucleus to cytoplasm is regulated by the acetylation of nuclear IFI16. Acetylated IFI16 proteins have been shown to be involved in IFN-β responses by recruiting STING to active TBK-1-IRF3 pathways (Ansari et al., 2015). In addition to DNA viruses, some intracellular bacteria, such as, *Listeria monocytogenes* (Hansen et al., 2014) and *Francisella novicida* (Storek et al., 2015), have also been shown to induce IFN-β expression through an IFI16 (IFI204) dependent pathway. We postulate that a similar mechanism in IFN-β release for IFI204 in response to *M. bovis* infection.

Induction of autophagy by many factors (such as: rapamycin and starvation) has been shown to inhibit mycobacterial survival and mediates mycobacterial clearance in infected macrophages by increasing acidification and maturation of mycobacterial phagosomes (Watson et al., 2012). *Mtb* can be targeted to ubiquitin-mediated selective autophagy by recognition of the extracellular bacterial DNA in the STING-dependent cytosolic pathway which is the downstream signal of IFI204 and cGAS, revealing a connection between cytosolic DNA sensor and autophagy in response to pathogens (Watson et al., 2012). The dsDNA sensor, cGAS has been shown to promote selective autophagy pathways during macrophage infection with *Mtb* (Collins et al., 2015; Watson et al., 2015), and this process is mediated by TBK1. Furthermore, the cGAS proteins can interact with Beclin-1, an autophagy protein, to shape the innate antimicrobial immune response (Liang et al., 2014). We hypothesize that IFI204 participates in autophagy activation through the STING-dependent phosphorylation of TBK-1 to inhibit *M. bovis* survival in macrophages.

To verify the role of IFI204 in IFN-β release and autophagy activation in macrophages during *M. bovis* infection, we investigated IFN-β release and IRF3 nuclear translocation during *M. bovis* infection of murine macrophages. We here show that this process depends on the acetylation of IFI204 protein followed by its translocation from nucleus to cytoplasm, and recruitment of STING to reduce IFN-β release by phosphorylation of TBK-1 and IRF3. Furthermore, knockdown of IFI204 decreased the autophagy marker LC3 expression and the co-localization between LC3 and *M. bovis*. Knockdown of IFI204 had no influence on the survival of *M. bovis* in macrophages *in vitro*. This may contribute to autophagy and IFN-β reduction by IFI204 which modulates intracellular replication of mycobacteria.

## Materials and methods

### Reagents

The mouse anti-Map-LC3 antibody (sc-376404), the rabbit polyclonal anti-mouse AIM2 antibody (sc-137967) and the goat polyclonal antibody TMEM173 (M-12) (sc-241049) were from Santa Cruz Biotechnology (Santa Cruz, CA, USA). The rabbit anti-mouse β-actin (AP0060) was obtained from Bioworld Technology (Nanjing, Jiangsu, China). The rabbit Anti-NAK/TBK-1 antibody (ab40676) and rabbit anti-NAK/TBK-1 (phosphor S172) antibody (ab109272) were obtained from Abcam (Cambridge, UK). The rabbit TMEM173 polyclonal antibody (19851-1-AP) and rabbit IRF3 polyclonal antibody (11312-1-AP) are from Protein Tech (Wuhan, Hubei, China). The phosphor-IRF3 (Ser396) (4D4G) and the Acetylated-Lysine Antibody (9441) were from Cell Signaling Technology (Boston, Mass, USA). The goat anti-rabbit secondary antibody, rabbit anti-mouse secondary antibody and donkey anti-goat secondary antibody were obtained from Santa Cruz Biotechnology, Beijing ZSGB Biotechnology and Beijing Cowin Biotechnology (Beijing, China), respectively. The BX-795 (S1274) was from Selleckchem. The Fast Protein Precipitation and Concentration Kit were purchased from Beyotime Institute of Biotechnology (Shanghai, China). Reagents and apparatus used in immunoblotting assays were obtained from Bio-Rad (Hercules, CA, USA). The mouse interferon-β ELISA kit (CSB-E04945m) was from Cusabio (Wuhan, Hubei, China).

### Cell culture

Mouse macrophage cell lines J774A.1 were obtained from Cell Culture Center, Xiehe Medical University (Beijing China), and were cultured as described before (Liu et al., 2016). Mouse bone marrow derived macrophages (BMDMs) were isolated from femurs of 6–8 week old female C57BL/6 mice as described previously (Liu et al., 2016), and cultured in circular cell culture dish (Corning, New York, USA) for 7 days in RPMI1640 (Hyclone, Logan, UT, USA) supplemented with 10 ng/ml M-CSF (Pepro Tech), 10% fetal bovine serum (FBS) (Gibco, Grand Island, NY, USA), 100 ug/ml streptomycin and 100 U/ml penicillin (Gibco).

### Bacterial culture and infections

Virulent *M. bovis* Beijing strain was obtained from China Institute of Veterinary Drug Control (CVCC, China). Bacteria were cultured from frozen stocks in 7H9 Middlebrook media (BD Biosciences) containing albumin- dextrose-catalase (ADC) enrichment solution and 0.05% Tween-80 (Difco) and grown to mid-logarithmic phase for 1 week at 37°C. J774A.1 and BMDMs were infected with *M. bovis* (MOI = 10:1) at 37°C with 5% CO_2_. Cells were washed three times with warm PBS to remove extracellular bacteria after 2 h. All experiments were performed in triplicate on three different occasions.

### Small interfering RNA (siRNA) transfections and treatments

Mouse IFI204-targeting siRNA oligonucleotide was obtained from Qiagen (Valencia, CA, USA). For siRNA transfection, cells were seeded in 24-well plates at a density of 1 × 10^5^ cells/well, and then transfected with siRNA oligonucleotides (50 nM) using 3 μL HiperFect Transfection Reagent (Qiagen, Valencia, CA, USA). The decrease in IFI204 expression was analyzed by qRT-PCR and western blot analysis.

### Immunohistochemistry (IHC)

The reagents for immunohistochemical (IHC) staining (TAHC03D) were obtained from BIOTnA (Sanming, Fujian, China), and the lung tissues from C57BL/6J mouse infected with *M. bovis* (CFU = 10^4^) for 4 weeks were stained as per manufacturers' instructions.

### Nuclear and cytoplasmic protein extraction

NE-PER Nuclear and Cytoplasmic Extraction Reagents (78833) was obtained from Thermo Scientific, the protein from nucleas and cytoplasm was extracted as per manufacturers' instructions.

### RNA isolation, complementary DNA synthesis and quantitative real-time polymerase chain reaction (PCR)

Total RNA was extracted using SV Total RNA Isolation System (Promega, Madison, WI, USA), and the concentration and integrity were detected by NanoDrop 2000 machine (Thermo SCIENTIFIC, Waltham, MA, USA). Reverse transcription of 100 ng RNA was performed by the Revert Aid first-strand cDNA synthesis Kit (Fermentas, Glen Burnie, MD, USA) according to the manufacturer's instructions, and the integrity and concentration of cDNA were detected by NanoDrop 2000 machine to confirm same amount of cDNA used for different samples. Quantitative PCR was performed using the DNA Engine Opticon TM2 fluorescence detection system (MJ Research Inc., Waltham, MA, USA) and SYBR Green Master Mix (Bio-Rad). The special gene primers pairs which cited from the publications (Yang et al., 2013) are shown in Table 1. Quantitative PCR data were analyzed using the comparative CT method (2^−ΔΔCT^) according to the application guidelines of fluorescence quantitative PCR from BIO-RAD. All samples were analyzed in triplicate.

**Table 1:** **Primers used for quantitative real-time PCR**.

**Gene**	**Forward primer (5′-3′)**	**Reverse primer (5′-3′)**
IFN-β	AAGAGTTACACTGCCTTTGCCATC	CACTGTCTGCTGGTGGAGTTCATC
IL-1β	AGAGCATCCAGCTTCAAATC	TCATCTCGGAGCCTGTAGTG
IL-10	GCTCTTACTGACTGGCATGAG	CGCAGCTCTAGGAGCATGTG
TNF-α	CCCTTCCTCCGATGGCTAC	CGCCTCCTTCTTGTTCTGG
β-actin	CCTTCTGACCCATTCCCACC	GCTTCTTTGCAGCTCCTTCG

### Immunobloting analysis

Total cell proteins were extracted using a protein extract kit (Fast Protein Precipitation and Concentration Kit, Wuhan Boster Biotech, Wuhan, China), and then were mixed with 5xSDS sample buffer. The mixtures were separated by different SDS-PAGE (8–15%) according to the molecular weight and analyzed by immunoblot as described previously (Liu et al., 2016). The image J software was used to analyze the intensity.

### ELISA

ELISA for IFN-β was performed with the macrophage cell culture supernatants by using mouse interferon-β ELISA kit as per manufacturers' instructions.

### Immunofluorescence assay

Ten microliter of a log-phase *M. bovis* culture were transferred into a 15-mL conical tube. Mycobacterial cultures were centrifuged at 3,000 rpm for 5 min, the supernatant fraction was carefully removed and the pellet washed twice with 10 mL of 1x PBS. This was centrifuged at 3,000 rpm for 5 min and the supernatant fraction removed and discarded. The pellet was resuspended in 1 mL of 1x PBS and the cell suspension transferred into a 1.5-mL microcentrifuge tube. 10 μl of Alexa 488 caboxylic acid succinimidyl ester stock solution was added to the tube to make a final concentration of 10 mg/ml. The tube was wrapped with foil and incubated at 37°C for 45–60 min on a shaker. Mycobacteria were pelleted by centrifugation at 10,000 rpm for 3 min at room temperature (RT). The supernatant was removed and washed twice with 1 mL of 1XPBS. The mycobacterial pellet was resuspended in 6 mL of complete DMEM. All reagents used for fixing, washing and blocking the cells were purchased from Beyotime (Beyotime Institute of Biotechnology, China). Cells were grown on poly-lysine-coated coverslips. After treatment, cells were fixed in an Immunol-staining fix solution (Beyotime) for 10 min at RT and washed three times for 5 min with PBS. Then they were blocked for 1 h at RT. Mouse anti-LC3B (1:200), rabbit anti-p-TBK-1 (1:200), rabbit anti-NAK (1:200), rabbit anti-IRF3 (1:200), rabbit anti-IFI204 (1:100) and goat anti-TMEM173 (1:50) were added, respectively, and incubated at 4°C overnight and washed three times with PBS for 5 min. Goat anti- rabbit IgG Alexa Fluor 594 (1:200), FITC-conjugated antibody (1:200), and donkey anti-goat IgG Alexa Flour 647 (1:200) were added and incubated at 37°C for 1 h and washed three times with PBS for 5 min. DAPI (1:10) was added and incubated at RT for 5 min and washed three times with PBS for 5 min. The macrophages were finally mounted with glycerin buffer and examined immediately with an OLYMPUS microscope.

### Co-IP

Cells were infected with *M. bovis* and washed twice with cold 1XPBS. Ice-cold RIPA or NP40 buffer with protease inhibitors was added and incubated at 4°C for 30 min on a rocker, to lyse the cells. Lysates were by micro-centrifuging at 13,200 rpm for 20 min in 4°C. The supernatant fraction was immediately transferred to a new centrifuge tube and the pellet was discarded. Cell lysate was incubated with 30 μl protein A or G agarose/sepharose beads (50%) at 4°C for 30 min on a rocker. Protein A or G beads were removed by centrifugation at 13,200 rpm at 4°C for 1 min and supernatant was transferred to a fresh centrifuge tube. A 30 μl aliquot od 50% protein A or G agarose/sepharose slurry was added with a specific antibody. After an overnight incubation at 4°C on a rocker the beads were washed 3–4 times for 10 min each with RIPA or NP40 buffer. Agarose/sepharose beads were resuspended in 30 μl 1X sample buffer and mixed gently. The agarose/sepharose beads were then boiled for 5min to dissociate the immune-complexes from the beads and spun down and the supernatants were used for SDS-PAGE and Western blot analysis.

### Colony-forming unit assay

J774A.1 and BMDMs cells were plated in 12-well plates (5 × 10^5^ cells/well). Cells were infected with 5 × 10^6^ mycobacteria per cells for 3 h at 37°C, washed three times with warm PBS. The CFUs were determined by plating 100 μL of serial dilutions onto plates containing Middlebrook 7H11 agar, supplemented with ADC enrichment solution and 0.05% Tween-80 (Difco).

### Statistical analysis

All assays were performed on two or three separate occasions in triplicate each time. Results are expressed as means ± S.D. All data comparisons were performed using one-way ANOVA followed by *post-hoc* Tukey's test or Student's *t*-test. SPSS software (Chicago, IL, USA) was used, and *P* < 0.05 was considered significant.

### Animal ethics statement

All protocols and procedures were performed according to the Chinese Regulations of Laboratory Animals—The Guidelines for the Care of Laboratory Animals (Ministry of Science and Technology of People's Republic of China) and Laboratory Animal Requirements of Environment and Housing Facilities (GB 14925–2010, National Laboratory Animal Standardization Technical Committee). The license number associated with their research protocol was 20110611–01 and the animal study proposal was approved by The Laboratory Animal Ethical Committee of China Agricultural University.

## Results

### *M. bovis* infection promotes IFN-β release and nuclear translocation of IRF3

**Figure 1:**
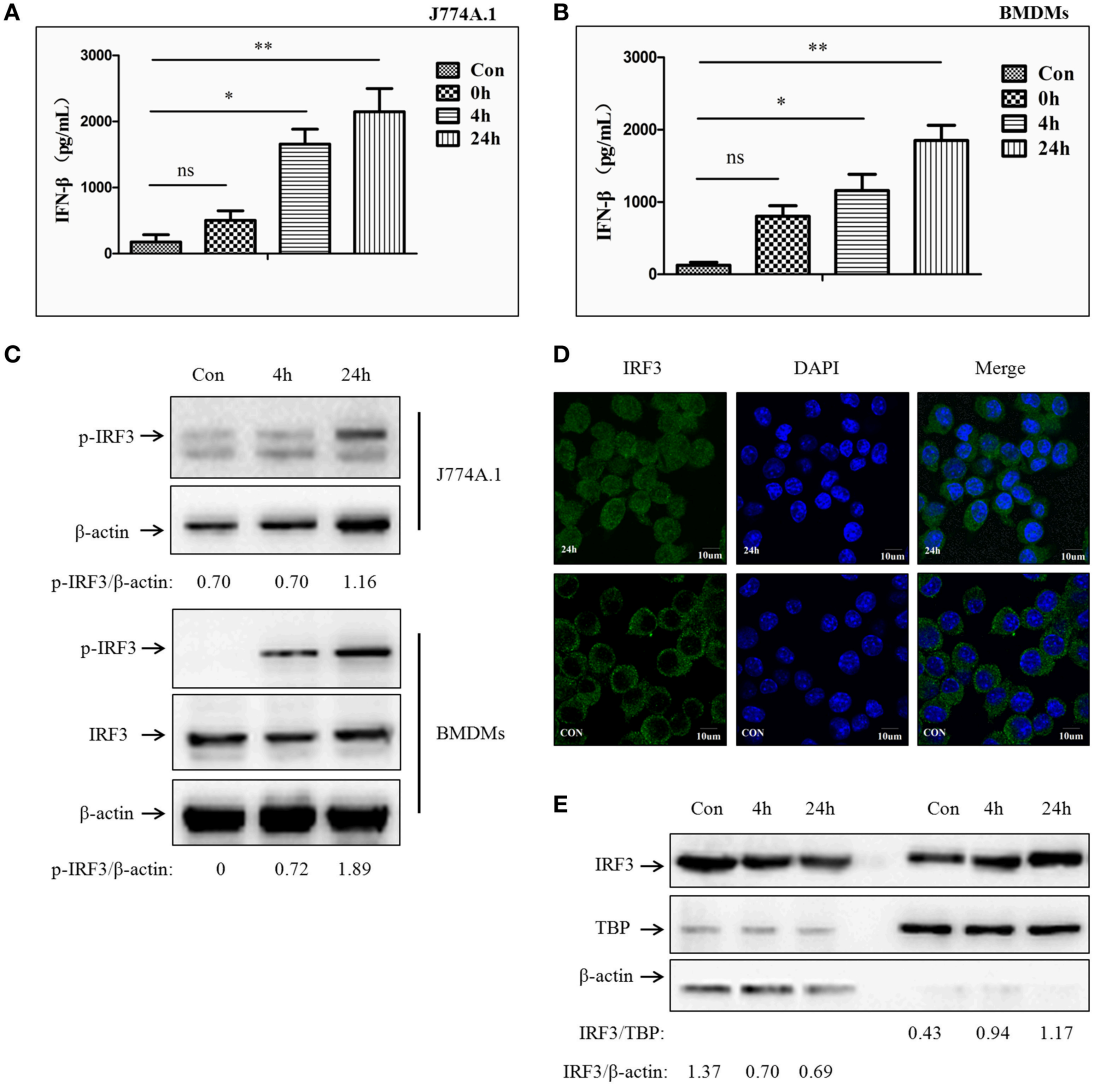
***M. bovis*** **infection promotes IFN-β release and nuclear translocation of IRF3. (A)** ELISA measurement of IFN-β protein released from J774A.1 cells infected with *M. bovis* (MOI 10) at different infection times (0, 4, 24 h). **(B)** ELISA measurement of IFN-β protein released from BMDMs cells infected with *M. bovis* (MOI 10) at different infection times (0, 4, 24 h). **(C)** J774A.1 and BMDMs cells were infected with *M. bovis*. The whole cells extracts were examined by immunobloting with anti-IRF3 and anti-p-IRF3 antibodies at different infection times (4, 24 h). The p-IRF3/β-actin ratios are shown below. **(D)** J774A.1 cells were infected with *M. bovis* for 24 h and immunostained with anti-IRF3 antibody (green). Cell nuclei were visualized by DAPI (blue). Scale bars, 10 μm. **(E)** J774A.1 cells were infected with *M. bovis*. Nuclear and cytosolic extracts were examined by immunobloting with anti-IRF3 antibody at different infection times (4, 24 h). These membranes were stripped and immunoblotted with anti-β-actin and anti-TATA binding protein (TBP) antibodies to examine the purity of nuclear (right) and cytoplasmic lysates (left), respectively. The IRF3/β-actin and IRF3/β-actin ratios are shown below. ^*^P < 0.05, ^**^P < 0.01.

Previous studies have demonstrated that type-I interferon could be induced by mycobacterium infection (Pandey et al., 2009; Manzanillo et al., 2012). STING-dependent IRF3 phosphorylation mediated by the cytosolic DNA sensor plays an important role in this process. Subsequently, the phosphorylated IRF3 (p-IRF3) translocates into the nucleus leading to IFN-β release (Dey et al., 2015). To examine whether murine macrophages respond similarly to *M. bovis*, J774A.1 cells and BMDMs were infected with a virulent strain of *M. bovis* isolated from cattle at a multiplicity of infection (MOI) of 10 and evaluated in a time course experiment (0, 4, and 24 h). Production of IFN-β was measured by ELISA (Figures 1A,B). We observed a time-dependent induction of IFN-β release. Western blot (WB) for total protein and phosphorylated counterparts were performed to investigate IRF3 responses during *M. bovis* infection. As expected, the IRF3 protein was phosphorylated during *M. bovis* infection (Figure 1C). Immunofluorescence (IF) microscopy showed that IRF3 translocated from cytoplasm to the nucleus at 24 h post-infection in J774A.1 cells (Figure 1D). Furthermore, the relative concentrations of IRF3 clearly increased in the nucleus and reduced in the cytoplasm of infected J774A.1 cells at 4 and 24 h-post-infection (Figure 1E). These results confirmed that IFN-β expression is induced in murine macrophages exposed to a virulent strain of *M. bovis*, and the release of IFN-β was dependent on IRF3 activation and its nuclear translocation.

### *M. bovis* infection increases the expression of IFI204 proteins

**Figure 2:**
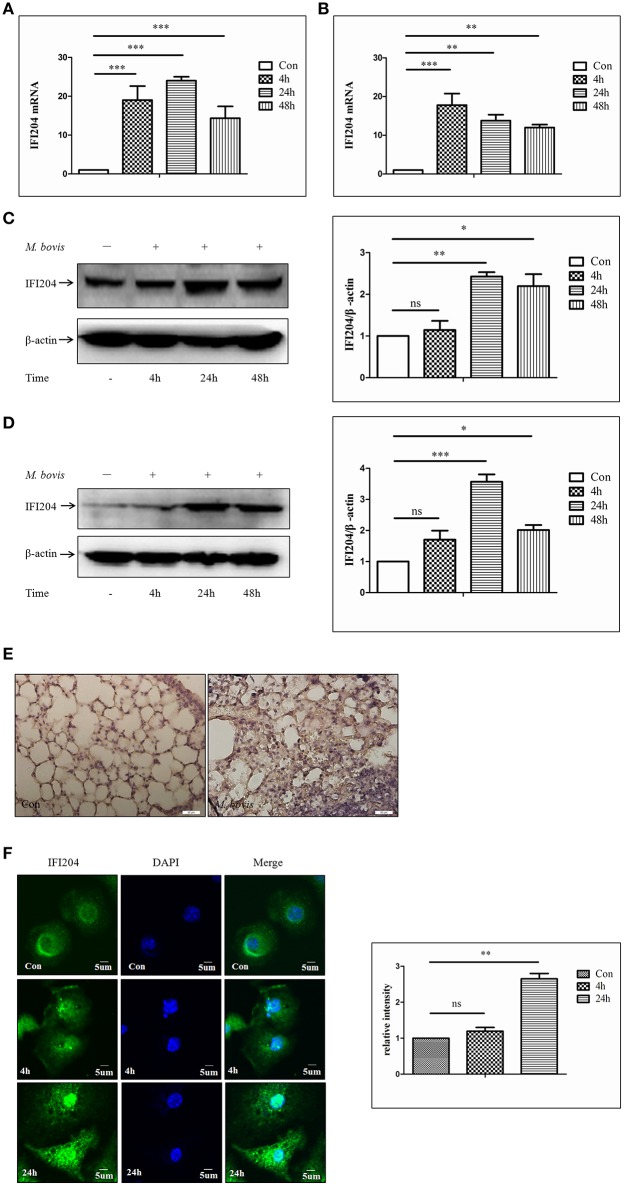
***M. bovis*** **Infection increases the expression of IFI204 proteins. (A,B)** Measurement of mRNA expression for IFI204 by q-PCR in J774A.1 **(A)** and BMDMs **(B)** cells infected with *M. bovis* (MOI 10) at different infection times (4 h, 24, and 48 h). **(C,D)** J774A.1 **(C)** and BMDMs **(D)** cells were infected with *M. bovis*. The whole cells extracts were examined by immunobloting with anti-IFI204 antibody. The ratios between IFI204 and β-actin were shown on right panel. **(E)** Immunohistochemistry analysis for IFI204 in a lung biopsy from a *M. bovis*-infected mouse. Scale bars, 20 μm. **(F)** BMDMs cells were infected with *M. bovis* for 24 h and immunostained with anti-IFI204 antibody (green) and relative fluorescence intensity was shown (right). Scale bar, 5 μm. Nearly 120 cells are used for the relative fluorescence intensity. The image J software was used for the analysis. ^*^P < 0.05; ^**^P < 0.01; ^***^P < 0.001.

To test whether IFI204 expression changes during *M. bovis* infection, we measured IFI204 mRNA level using q-PCR, at different infection time points. We observed that the mRNA expression of IFI204 was upregulated in both J774A.1 (Figure 2A) and BMDMs (Figure 2B). IFI204 protein showed a sustained increase in both cell types and peaked at 24 h post-infection (Figures 2C,D). The protein production of IFI204 showed a behind its mRNA expression may attribute to the asynchronous expression of the gene and protein. The results also showed the gene expression of IFI204 decreased after 48 h in J774A.1 but at 24 and 48 h in BMDM cells. One reason for this difference may be related to more rapid proliferation by J774A.1 cells. Another factor is M-CSF within the myeloid cell culture medium affects the IFI204 expression (Dauffy et al., 2006). IFI204 expression in the lung lesions of mice infected with *M. bovis* showed significantly higher IFI204 expression in the sections with granulomas (Figure 2E). We then demonstrated that IFI204 was present in both the cytoplasm and the nucleus and the concentrations of IFI204 were higher in the nucleus (Figure 2F). Furthermore, we showed that the relative intensity of IFI204 was enhanced in both cytoplasm and nucleus at 24-h post-infection. Taken together, IFI204 is induced by *M. bovis* infection in murine macrophages. It was also expressed in the lungs of *M. bovis* infected mice.

### IFI204 is required for IFN-β release in murine macrophages upon *M. bovis* infection

**Figure 3:**
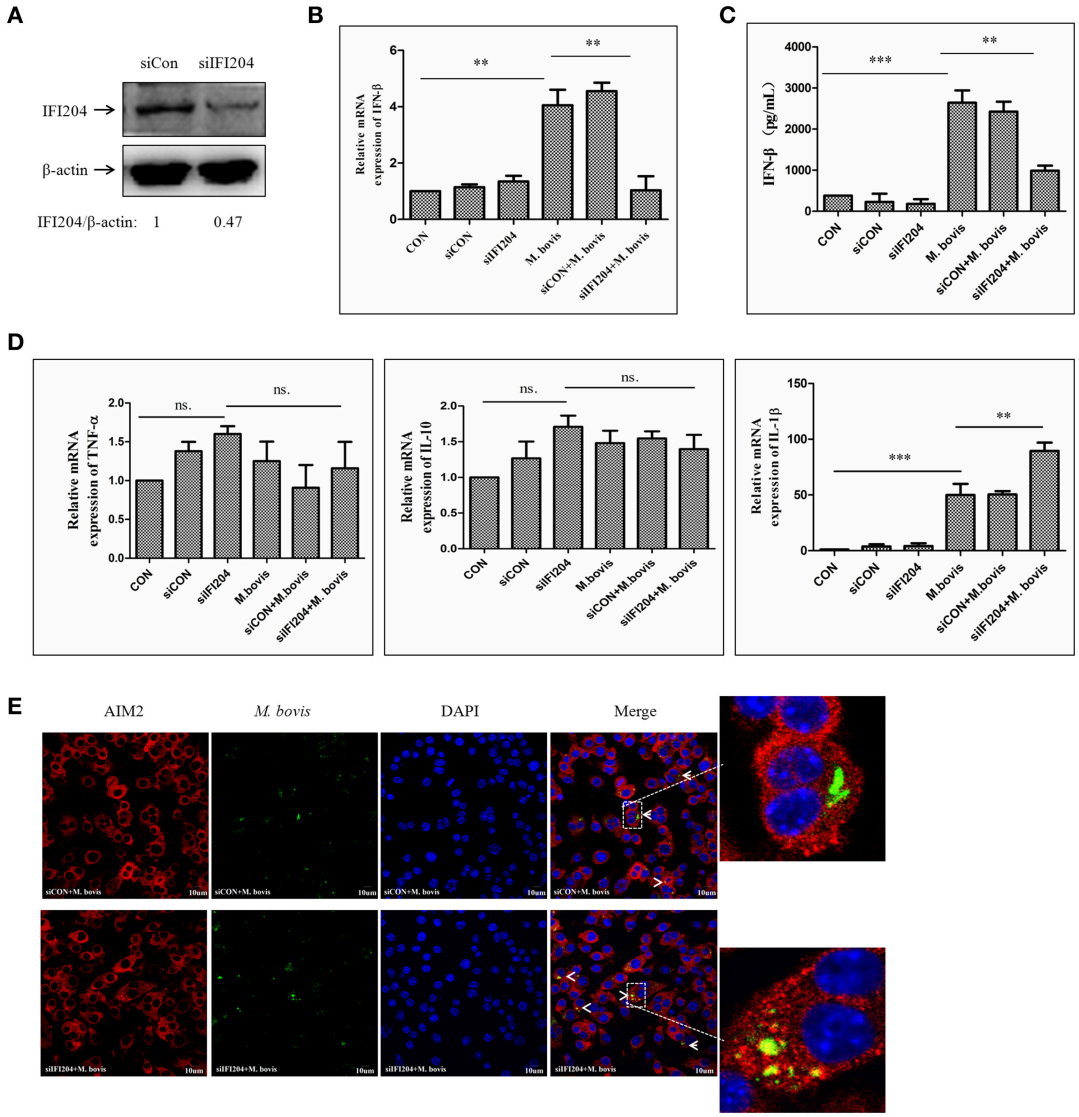
**IFI204 is required for IFN-β release in murine macrophages upon *M. bovis* infection. (A)** Western blot of the expression of IFI204 in BMDMs transfected with non-targeting siRNA (siCon), or IFI204-targeting siRNA (siIFI204). The AIM2/β-actin ratios are shown below. **(B)** Measurement of mRNA expression for IFN-β by q-PCR in BMDMs cells transfected with siCon, or siIFI204 and then infected 24 h with *M. bovis* (MOI 10). **(C)** ELISA measurement of the protein expression of IFN-β in BMDMs cells transfected with siCon, or siIFI204 and then infected 24 h with *M. bovis* (MOI 10). **(D)** The mRNA expression of TNF-α, IL-1β and IL-10 by q-PCR in BMDMs cells transfected with siCon, or siIFI204 and then infected 24 h with *M. bovis* (MOI 10). **(E)** J774A.1 cells were infected with Alexa-488 Carboxylic Acid (green) labeled *M. bovis* for 24 h and immunostained with anti-AIM2 antibody (red), Scale bar, 10 μm. ^**^P < 0.01; ^***^P < 0.001.

To examine the role of IFI204 in IFN-β release in response to *M. bovis* infection, we applied IFI204 specific small interfering RNA (siRNA) to knock down the expression of IFI204 in the BMDM infection model. The results showed that IFI204 expression decreased by 55 percent post-blocking (Figure 3A). IFN-β expression measured by qPCR and ELISA showed that both mRNA (Figure 3B) and protein (Figure 3C) expression decreased in IFI204 knock-down groups. Gene expression of TNF-α and IL-10 did not show any significant changes but an increase in IL-1β levels were observed (Figure 3D). We have recently shown that AIM2 plays a critical role in IL-1β release and it inhibits the STING-dependent pathway during *M. bovis* infection in macrophages (Liu et al., 2016). We hypothesized that IFI204 conjugated competitively with *M. bovis* to inhibit the AIM2-dependent IL-1β production. To test this hypothesis, we examined the co-localization of *M. bovis* and AIM2 after IFI204 knocked down. *M. bovis*-AIM2 co-localization increased moderately (Figure 3E). Taken together, these results suggest that IFI204 plays an important role in IFN-β release during *M. bovis* infection in murine macrophage model.

### Acetylated IFI204 co-localizes with *M. bovis* in the cytoplasm and recruits STING to activate IFN-β release

**Figure 4:**
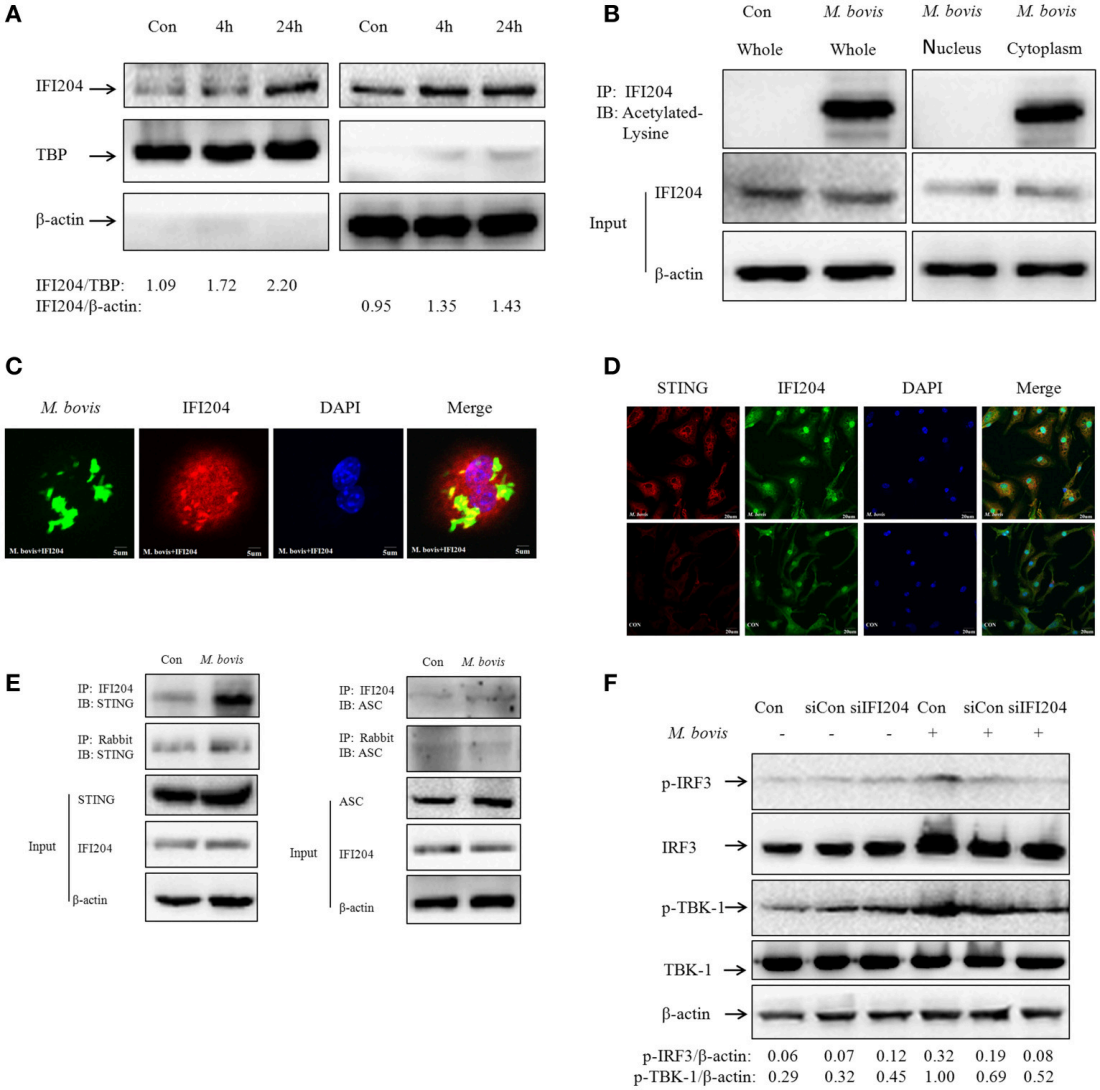
**Acetylated IFI204 co-localizes with *M. bovis* in the cytoplasm and recruits STING to activate IFN-β release. (A)** J774A.1 cells were infected with *M. bovis* and nuclear (left panel) and cytosolic extracts (right panel) were examined by immunobloting with anti-IFI204 antibody. These membranes were stripped and immunoblotted with anti-β-actin and anti-TBP antibodies to examine the purity of nuclear and cytoplasmic lysates, respectively. The ratios of IFI204/TBP and IFI204/β-actin are shown below. **(B)** J774A.1 cells were infected with *M. bovis* for 24 h. The whole cells extracts (left band) or nuclear and cytosolic extracts (right band) were immunoprecipitated with IFI204 antibody; immunoblots of immune complexes with Acetylated Lysine antibody and inputs were probed with IFI204 and β-actin antibodies. **(C)** J774A.1 cells were infected with Alexa-488 Carboxylic Acid (green) labeled *M. bovis* for 24 h and immunostained with anti-IFI204 antibody (red). Scale bar, 5 μm. Fifty two cells showed IFI204-positive mycobacteria in approximately 120 cells. The image J software was used for the analysis. **(D)** BMDMs cells were infected with *M. bovis* for 24 h and immunostained with anti-STING (red) and anti-IFI204 (green) antibody to observe the co-localization between STING and IFI204. Cell nuclei were visualized by DAPI (blue). Scale bar, 20 μm. **(E)** J774A.1 cells were infected with *M. bovis* for 24 h. The whole cells extracts were immunoprecipitated with IFI204 antibody or rabbit antibody; immunoblots of immune complexes and inputs were probed with IFI204, STING and β-actin antibodies (left band) or IFI204, ASC and β-actin antibodies (right band). **(F)** BMDMs cells transfected with siCon, or siIFI204 and then infected 24 h with *M. bovis* (MOI 10). Whole-cell extracts were analyzed with Abs against p-TBK1, total TBK1, p-IRF3, total IRF3, and β-actin. The p-IRF3/β-actin and p-TBK-1/β-actin ratios are shown below.

As shown in Figure 2F, IFI204 expression increased in the cytoplasm and the nucleus during *M. bovis* infection. Furthermore, the IFI204 protein levels in the respective cellular compartments were confirmed by WB (Figure 4A). Results show that the expression in both cellular sub-compartments increased in a time dependent manner during macrophage infection. The translocation of IFI16 from cytoplasm to nucleus is regulated by the acetylation of nuclear IFI16 during viral infections (Ansari et al., 2015). We found that IFI204 was acetylated in macrophages during *M. bovis* infection and the acetylated protein was distributed in cytoplasm but not in the nucleus (Figure 4B). We next demonstrated that cytosolic IFI204 co-localized with *M. bovis* (Figure 4C) in 42% infected macrophages. STING is the adapter protein for IFI16 which is known to be required for TBK1-IRF3-dependent IFN-β responses to viruses and DNA (Unterholzner et al., 2010). To identify if STING molecule was recruited by Ifi204 during *M. bovis* infection, we used IF to observe the co-localization between them at 24h post-infection in BMDMs (Figure 4D). Co-immunoprecipitation (co-IP) experiments showed that STING was present in IFI204 immunoprecipitates, suggesting existence of a IFI204-STING conjugate molecule in J774A.1 cells (Figure 4E, left column). No interaction was observed between ASC and IFI204 in the infected cells (Figure 4E, right column). This result further confirmed our hypothesis that IFI204 conjugates competitively with *M. bovis* to inhibit the AIM2-dependent IL-1β production. To examine the influence of IFI204 on downstream signaling pathway, we performed siRNA to down-regulate IFI204 expression and then tested the phosphorylation of TBK1 and IRF3 by WB in J774A.1-*M. bovis* infection model. We observed a decrease in STING-dependent signaling molecules (Figure 4F). Taken together, we conclude that IFI204 first undergoes acetylation and then translocates from nucleus into cytoplasm to recruit STING for activation of TBK1-dependent IRF3 nuclear translocation and IFN-β release.

### IFI204 is required for LC3 co-localization with *M. bovis*

**Figure 5:**
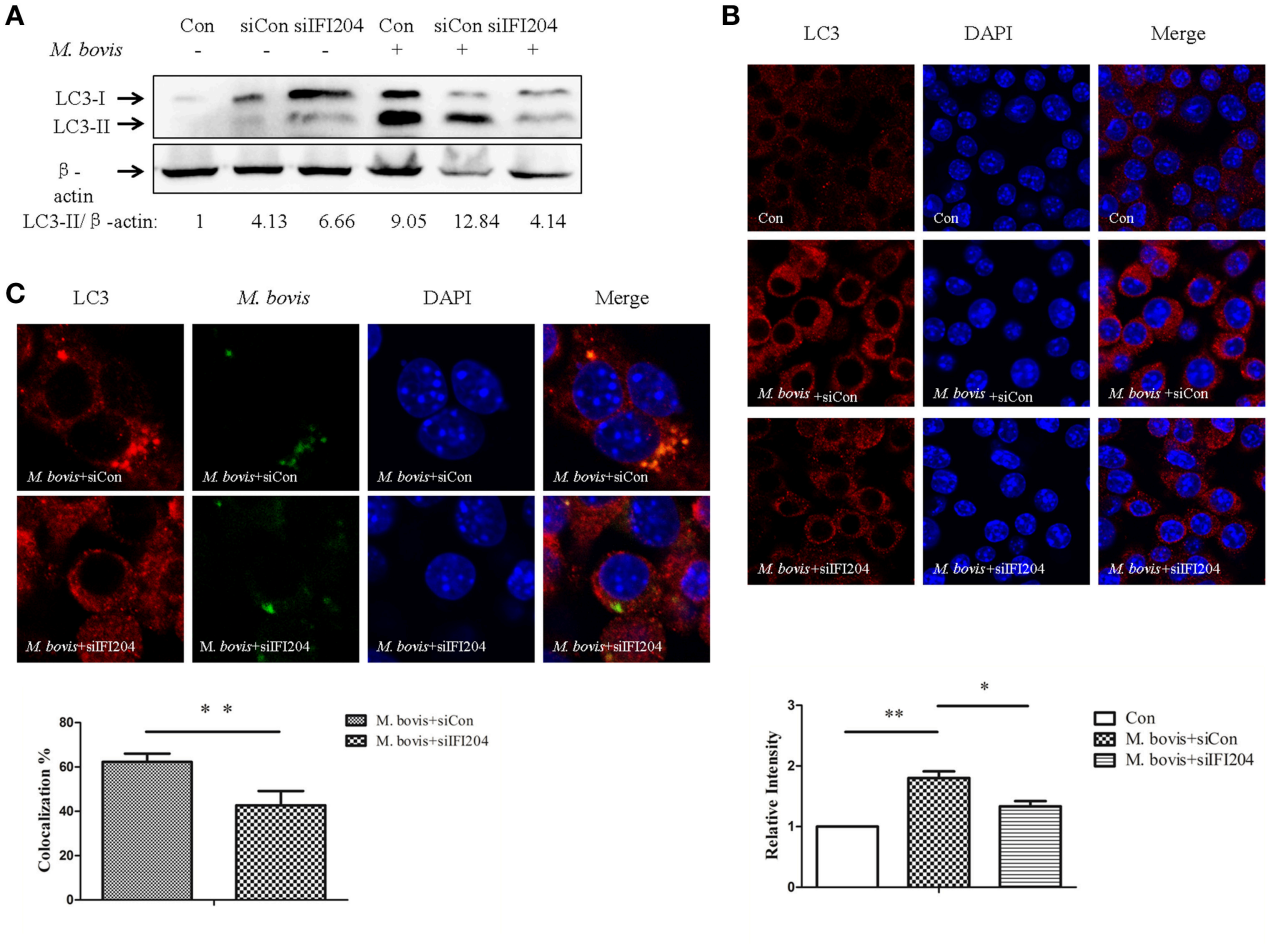
**IFI204 is required for LC3 co-localization with *M. bovis*. (A)** BMDMs cells transfected with siCon, or siIFI204 and then infected 24 h with *M. bovis* (MOI 10). Whole-cell extracts were analyzed with Abs against LC3-II and β-actin. The LC3-II/β-actin ratios are shown below. **(B)** J774A.1 cells were transfected with siCon, or siIFI204 and then infected with *M. bovis* for 24 h and immunostained with anti-IFI204 antibody (green) and relative fluorescence intensity was shown (below). Scale bar, 5 μm. Nearly 120 cells are used for the relative fluorescence intensity. The image J software was used for the analysis. **(C)** J774A.1 cells were transfected with siCon, or siIFI204 and then infected with Alexa-488 Carboxylic Acid (green) labeled *M. bovis* for immunostained with anti-LC3 antibody (red) and the proportion of LC3-positive mycobacteria is shown (below), Scale bar, 5 μm. Approximately 120 cells are used to calculate the co-localization proportion. The image J software was used for the analysis. ^*^P < 0.05, ^**^P < 0.01.

TBK1 has been shown to play an important role in autophagy activation during cytosolic bacterial infection (Pilli et al., 2012). The dsDNA sensor cGAS has been shown to be associated with induction of autophagy and co-localization between LC3 and *Mtb* in macrophages (Watson et al., 2015). To test the role of IFI204 in autophagy, we examined the LC3 expression in BMDMs transfected with IFI204 targeting siRNA and then infected with *M. bovis*. We observed that LC3-IIexpression decreased when IFI204 was knocked down (Figure 5A). Similar results were observed when LC3-puncta were counted (Figure 5B). Next, we tested whether IFI204 also had an effect on the co-localization between *M. bovis* and LC3. Absence of IFI204 significantly reduced the association of LC3 with *M. bovis* (Figure 5C).

### IFI204 modulates the balance of *M. bovis* replication in macrophages

**Figure 6:**
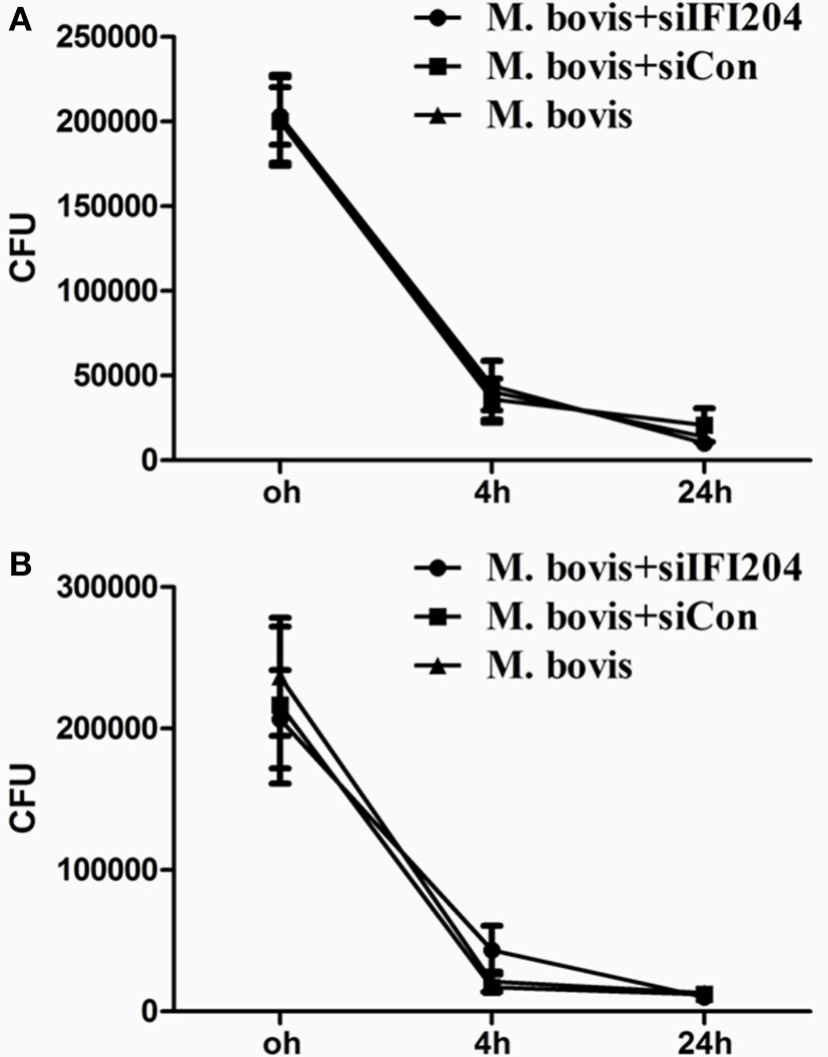
**IFI204 modulates the balance of *M. bovis* replication in macrophages. (A,B)** Colony-Forming Units (CFU) from J774A.1 macrophages **(A)** and BMDMs cells **(B)** both transfected with siCon or siIFI204 and then infected with *M. bovis* (MOI = 10).

Type I interferons (IFN) are pleiotropic proteins with anti-proliferative, antiviral, and immunomodulatory activities. It is widely believed that IFN-β is an adverse factor for host survival, but advantageous to mycobacterial survival. Multiple investigations have demonstrated that induction of autophagy inhibits mycobacterium replication in macrophages. Furthermore, cGAS, which has been shown to induce type-I interferon and activate autophagy during *Mtb* infection, is necessary for mycobacterium control. To examine the role of IFI204 in *M. bovis* survival, we infected BMDMs and J774A.1 deficient in IFI204 and quantified intracellular mycobacterium replication (Figure 6). No significant differences were observed in *M. bovis* replication between knockdown and untreated cells. These results suggest an important role of IFI204 in IFN-β release and autophagy activation in murine macrophages during *M. bovis* infection.

## Discussion

In this investigation, we show that IFI204 plays a central role in IFN-β induction and autophagy activation in murine macrophages infected with *M. bovis*. The nuclear IFI204 was acetylated and then translocated into cytoplasm to recruit the adaptor protein STING for IRF3-dependent IFN-β release and TBK-1-LC3 associated autophagy during *M. bovis* infection. Our results provide concrete evidence for a role of IFI204 in *M. bovis* survival. This molecule has also been shown to contribute toward cytosolic surveillance pathway (CSP) activation (Manzanillo et al., 2012). TBK-1 is required for autophagic elimination of mycobacteria in macrophages and further research shows that c-GAS associated selective autophagy needs p-TBK-1 (Pilli et al., 2012; Watson et al., 2015). Our results show IFI204 which required for LC3 co-localization with *M. bovis* is needed for the phosphorylation of TBK-1. Mechanism of acetylation of IFI204 is still unknown. In addition to cGAS and AIM2 (Yang et al., 2013; Majlessi and Brosch, 2015), we here established a role of another DNA sensor, IFI204, during mycobacterium infection.

Several members of the ds-DNA sensors have also been proposed to activate the IFN-β release or IL-1β production during *Mtb* infection, including cGAS and IFI204 or AIM2 (Manzanillo et al., 2012; Yang et al., 2013; Collins et al., 2015). Except cGAS, AIM2 and IFI204 are both AIM2-like-receptors (ALRs) characterized by a pyrin signaling domain and a DNA-binding HIN domain. Although AIM2 is well-defined as the key DNA sensor that activates the inflammasome, and cGAS itself is also well-defined as the key DNA sensor for type-I interferon release, but the function of IFI204 and other ALRs appear to function differently. In this study, we demonstrated that ALRs are essential for activation of the interferon response to intracellular DNA (Gray et al., 2016). IFI16 has been shown to be required for the IFN response to HCMV using siRNA approaches (Ansari et al., 2015). On the other hand, studies have also shown that IFI16 is dispensable for the IFN response to HCMV Infection in knock out cell models (Gray et al., 2016). These differences in results may be attributed to the differences between knock-out vs. knock-down approaches used in these studies. In the present study, we used siRNA approaches for IFI204 silencing and showed similar results to those performed with shRNA treatment (Manzanillo et al., 2012).

We have previously shown the potential competitive relationship between AIM2 and other DNA sensors involved in STING dependent pathway (Liu et al., 2016). In this study, we found that IFI204 knockdown has an effect on AIM2-dependent IL-1β release confirming the possibility that ALRs mechanistically interact and co-regulate function (Gray et al., 2016). TBK-1 has been shown to promote autophagy-mediated antimicrobial defense by controlling autophagosome maturation (Pilli et al., 2012). Furthermore, cGAS has been shown to play an important role in recruiting these selective autophagy markers to intracellular *Mtb* by p-TBK1 (Watson et al., 2015). Inducing autophagy by exogenous agents inhibits pathogen survival. Mice with an Atg5 (autophagy-related protein 5) deletion in the myeloid lineage are more susceptible to mycobacterium infection (Castillo et al., 2012). These results illustrate the important role of autophagy in controlling mycobacterial damage to the host. Here, we demonstrated that IFI204 dependent p-TBK1 also participates in recruiting autophagy marker LC3.

Some investigations demonstrated that the cytosolic bacterial genomic DNA plays an important role in autophagy induction, AIM2 inflammasome activation and type-I interferon reactions (Manzanillo et al., 2012; Saiga et al., 2012; Watson et al., 2012). While some investigators have shown the mitochondrial stress and cytosolic mtDNA contribute toward IFN-β induction (Wiens and Ernst, 2016), there is paucity of definitive data on specific molecules involved in the acetylation and translocation of IFI204. Our studies define the mechanism behind the acetylation of IFI204 during *M. bovis* infection in a time course infection model. Whether, other members of ALR genes in humans (PYHIN1 and MNDA) which also have PYHIN and HIN domains may play a role in innate immune response during mycobacterial infections, needs further investigation.

Taken together, understanding the mechanism of IFN-β induction and autophagy activation by *M. bovis* infection may facilitate the development of host-directed TB treatments. IFI204, a member of ALRs, which participates in IFN-β release and autophagy induction during mycobacterial infection, supplies another consideration for drug targets screening and drug therapy.

## Author contributions

LC wrote the manuscript. LY, DZ, and XZ designed experiments. LC and SX performed experiments. LQ assisted to complete experiments. SS rewrote and edited the manuscript.

## Funding

This work was supported by the MoSTRCUK international cooperation project (Project No. 2013DFG32500), National Natural Science Foundation of China (Project No.31572487), Funding of State Key Lab of Agrobiotechnology (Project No. 2012SKLAB06-14). 2015 CAU Foreign Experts Major Projects (Project No: 2012z018). High-end Foreign Experts Recruitment Program (Project No: GDW20151100036).

### Conflict of interest statement

The authors declare that the research was conducted in the absence of any commercial or financial relationships that could be construed as a potential conflict of interest.
